# Combining de novo and reference-guided assembly with scaffold_builder

**DOI:** 10.1186/1751-0473-8-23

**Published:** 2013-11-22

**Authors:** Genivaldo GZ Silva, Bas E Dutilh, T David Matthews, Keri Elkins, Robert Schmieder, Elizabeth A Dinsdale, Robert A Edwards

**Affiliations:** 1 Computational Science Research Center, San Diego State University, San Diego, CA 92182, USA; 2 Department of Computer Science, San Diego State University, 5500 Campanile Drive, San Diego, CA 92182, USA; 3 Department of Biology, San Diego State University, 5500 Campanile Drive, San Diego, CA 92182, USA; 4 Centre for Molecular and Biomolecular Informatics, Nijmegen Centre for Molecular Life Sciences, Radboud University Medical Centre, Geert Grooteplein 28, 6525 GA, Nijmegen, the Netherlands; 5 Department of Marine Biology, Institute of Biology, Federal University of Rio de Janeiro, Rio de Janeiro, Brazil; 6 Division of Mathematics and Computer Science, Argonne National Laboratory, 9700 S. Cass Ave, Argonne, IL 60439, USA

**Keywords:** Scaffolding, *De novo* assembly, Reference genome, Genome sequencing, Next generation sequencing, *Salmonella enterica* serovar typhimurium, *Salmonella* typhimurium

## Abstract

Genome sequencing has become routine, however genome assembly still remains a challenge despite the computational advances in the last decade. In particular, the abundance of repeat elements in genomes makes it difficult to assemble them into a single complete sequence. Identical repeats shorter than the average read length can generally be assembled without issue. However, longer repeats such as ribosomal RNA operons cannot be accurately assembled using existing tools. The application *Scaffold_builder* was designed to generate scaffolds – super contigs of sequences joined by N-bases – based on the similarity to a closely related reference sequence. This is independent of mate-pair information and can be used complementarily for genome assembly, e.g. when mate-pairs are not available or have already been exploited. *Scaffold_builder* was evaluated using simulated pyrosequencing reads of the bacterial genomes *Escherichia coli* 042, *Lactobacillus salivarius* UCC118 and *Salmonella enterica* subsp. enterica serovar Typhi str. P-stx-12. Moreover, we sequenced two genomes from *Salmonella enterica* serovar Typhimurium LT2 G455 and *Salmonella enterica* serovar Typhimurium SDT1291 and show that *Scaffold_builder* decreases the number of contig sequences by 53% while more than doubling their average length. *Scaffold_builder* is written in Python and is available at http://edwards.sdsu.edu/scaffold_builder. A web-based implementation is additionally provided to allow users to submit a reference genome and a set of contigs to be scaffolded.

## Background

Second generation sequencing remains the most cost-effective and readily available technique for complete genome sequencing. While read lengths are increasing, assembly and scaffolding of complete genome sequences often remains a challenge [1]. Paired-end sequencing can greatly improve this by creating scaffolds [2], but if paired-end information is not available or has been exhausted, the similarity provided by a closely related reference genome can provide independent information to assist with scaffolding of the contigs [3]. Some assemblers, MIRA [4] for example, can create a reference-based assembly, where the genome is scaffolded during the assembly process, and impose the complete genome structure of the reference on the assembly [5,6]. While this restriction may not be problematic for genomes with low rearrangement rates, some bacterial genomes are highly plastic with mobile regions that can be located in different genomic locations even in closely related species [3,7]. In addition to mobile genetic elements, bacterial genomes frequently have large-scale rearrangements via recombination between multicopy sequences such as IS elements and *rrn* operons. These rearrangements decrease synteny between related genomes by either inverting or translocating the intervening region between the multicopy sequences [8,9].

Most scaffolding programs that are currently available use the information provided by mate-pair sequencing to combine contigs into longer scaffolds [2,10]. Also, there is software for manual genome scaffolding [11] based on ordering the contigs. Here, we present the program *Scaffold_builder* that provides a complementary approach, exploiting the homology of a reference genome to order contigs and build scaffolds. After composing an initial *de novo* assembly from reads and possible paired-end data, a scaffold of the contigs is built using a reference genome. Thus, we accept the contig sequences in regions where the *de novo* assembly is certain, and allow the reference genome to add structure to the composed sequence by ordering and orienting the contigs. An additional feature of *Scaffold_builder* compared to tools like CONTIGuator [12], Projector 2 [13], and ABACAS [14], e-RGA [15] which take a similar approach of contig mapping, is that sequential contigs can be merged if their terminal sequences are highly similar. In such cases, the reference genome helps resolve cases where the *de novo* assembly program broke the sequence into separate contigs, e.g. because the overlapping region was too short or because the assembly was ambiguous. Scaffolds provide a better insight into the sequencing coverage, and generate a more accurate estimation of the number and sizes of the remaining gaps in the sequence. Moreover, the map of the scaffolded contigs to the reference genome allows an exploration of whether those gaps lie in toxic regions that may prevent cloning, or in complex regions that may hinder accurate sequencing with one or more current sequencing approaches. Thus, the scaffolds can direct closing efforts, the most difficult part of completing microbial genomes.

## Methods

### Real data

Two laboratory derived derivatives of *Salmonella enterica* serovar Typhimurium LT2 (*S.* Typhimurium LT2), SDT1291 and G455 were sequenced on the 454 GS FLX Titanium. Sequences were deposited in the Sequence Read Archive (ERP000942). An initial assembly was constructed using Newbler 2.7 [16] with default parameters. All contigs were used for scaffolding. The complete *S.* Typhimurium LT2 genome sequence (NC_003197) in the SEED database [17] was used as reference.

### Simulated data

Simulated reads were sampled from three complete bacterial genomes: *Escherichia coli* 042 (FN554766), *Lactobacillus salivarius* UCC118 (NC_007929) and *Salmonella enterica* subsp. *enterica* sv Typhi P-stx-12 (NC_016832) using GemSIM 1.6 [18]. 400,000 reads with a length of 395 nt ± 116 nt were generated per genome using the supplied error model for Roche/454; these simulated reads were assembled using Newbler 2.7 [16] as above. Moreover, 1,600,000 paired 2 × 101 nt reads were generated per genome using supplied error model for Illumina GA IIx with TrueSeq SBS Kit v5‒GA and assembled using MIRA 3.19 [4]. Ten simulated datasets were generated for each strain.

### Scaffolding

**Figure 1:**
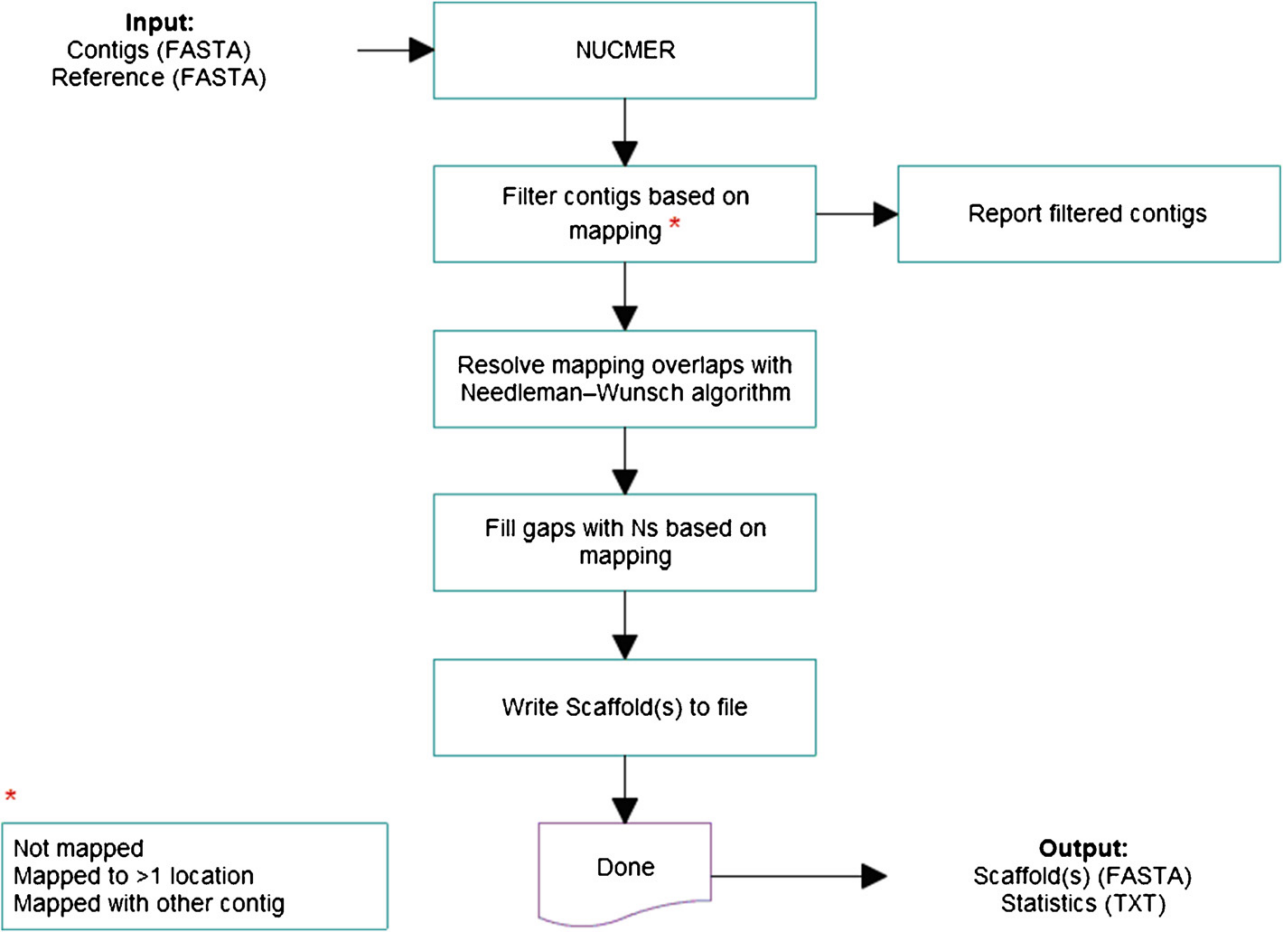
Flow diagram of the Scaffold_builder script.

The *Scaffold_builder* program performs several analysis steps (Figure 1). First, Nucmer (with default parameters) is run to map contigs to the reference genome and the hits are parsed with Show-coords [19]. Contigs mapped to more than one location over at least 95% of their length (a default value) are considered ambiguously mapped and reported separately. Then, *Scaffold_builder* uses the location of the longest hit to place the entire contig, while extending any unmapped “overhangs” along the reference. Although this potentially extends the alignment beyond similar region, we trust the sequence of the contig to be correct. Any contig that aligns entirely within a region that already contains a longer contig is not scaffolded and reported separately (as a duplicate region). The algorithm then proceeds along the reference sequence and inserts an appropriate number of Ns between every pair of non-overlapping contigs. For contigs that are mapped with an overlap, *Scaffold_builder* checks whether those contigs could be joined, broken, or placed end-to-end, by determining the sequence identity between their terminal regions using the Needleman–Wunsch algorithm [20]. Initially, the length of the terminal sequences tested for 300 nt of similarity, or less if the overlapping region is shorter. Then, the program elongates these regions in increments of 10 nt and re-aligns them to a maximum of 400 nt, testing if the similarity between the terminal sequences exceeds the minimum identity threshold of 80%. If the minimum identity is not reached, then *Scaffold_builder* either joins the contigs end-to-end (default) or breaks the scaffold. All the values mentioned above are default values and can be optionally adjusted by the user.

### Output

The program outputs a log file with all the decisions that were made, a map of the original and scaffolded locations of the sequence against the reference genome, and a summary of the statistics associated with the assembly before and after scaffolding.

### Web-based version

As an alternative to the command line version of the program, we have created a user-friendly web version of *Scaffold_builder* which provides a tutorial and example of input and output file. The web server is available at http://edwards.sdsu.edu/scaffold_builder.

## Results and discussion

*Scaffold_builder* is a versatile tool that allows ordering and merging of *de novo* assembled contigs by using a homologous reference genome. Using three simulated datasets, as well as two newly sequenced *S.* Typhimurium LT2 genomes, we show that *Scaffold_builder* greatly reduced the number of contigs of the draft genomes. As shown in Additional file 1: Table S1, the number of contigs was decreased by 53% ± 12% while increasing their average length by 114% ± 65%.

An average of 38% ± 13% of the contigs were not scaffolded, including sequences that could not be mapped, sequences that mapped in a location that was occupied by another contig with a longer hit, and contigs that were mapped ambiguously. Notably, an average of more than 63% ± 31% of contigs that overlapped after mapping to the reference sequence could be merged using the default identity threshold of 80% (see Additional file 1: Table S1). The average length of these merged regions was 56 nt ± 24 nt. Although these short regions could not unambiguously be assembled by the Newbler or MIRA assembler, the high sequence identity combined with the homology of the reference genome nevertheless enabled *Scaffold_builder* to join them into a single scaffold.

Genes that overlapped with the break points between contigs include *rrn* operons, transposases and other known multi-copy genes (Additional file 2: Table S2). This illustrates that a reference sequence provides the structure needed to bridge many of the repeat regions that the *de novo* assembler was unable to join.

The increase in sequence length obtained by using *Scaffold_builder* compares well with the results obtained with CONTIGuator [12] when using similar mapping parameters (CONTIGuator uses blastn [21] to map the contigs). In all of the simulated and real cases examined, *Scaffold_builder* scaffolded the same number or more sequences than CONTIGuator (Additional file 3: Table S3).

Although *Scaffold_builder* was written and tested using bacterial genomes, the tool can also be used with smaller or larger genomes. For eukaryotic genomes Nucmer requires more memory; for example, for the largest human chromosome Nucmer requires 15.4 bytes per base pair [19]. *Scaffold_builder* has been used to create scaffolds of the 3.1 Gbp genome of the California Sea lion, *Zalophus californianus*, that was scaffolded against its closest relative, the dog *Canis familiaris*[22]. *Scaffold_builder* greatly extended the length of the contigs compared with the initial assembly.

Even though the tool was tested with second-generation sequencing data, *Scaffold_builder* does not rely on a particular platform. The third-generation sequencing platforms, e.g. PacBio RS, provide longer reads and contigs than the previous generations [23]. Long contigs facilitate the mapping, ordering and scaffolding of the sequences and reduces the number of ambiguous sequences.

### Limitations

Scaffold_builder depends on an available genome sequence of a closely related organism. Mapping success depends on sequence similarity [24]. One limitation of Scaffold_builder is the inability to detect large-scale genomic rearrangements relative to the reference if the end points of the rearrangements fall within contig gaps. Multi-copy sequences such as rRNA operons may more frequent in rearrangement breakpoints due to similar recombination, and they are also more difficult to assemble due to ambiguous read mapping. The presence of large-scale rearrangements relative to the reference can be resolved by either using mate-pair sequencing or long-read sequencing across the gaps, or by using PCR to determine the correct sequences flanking the gap [25].

*Scaffold_builder* tries to join contigs if their overlapping region is highly similar in sequence. In order to test the program limitations, we selected one simulated dataset of *Lactobacillus salivarius* UCC118, and using Mauve [26] we evaluated 30 sequential contig pairs that exceeded the identity threshold of 80% and were merged. Only 1 pair of contigs was joined incorrectly.

Contigs mapped to more than one location over at least 95% of their length are not scaffolded. These contigs are labeled as ambiguous in the output file, and probably the result of a duplicated region in the reference genome. Conversely, contigs that are mapped to the reference within a region that already contains a longer contig are duplicated regions in the query genome, and also reported separately.

## Conclusions

Here we present *Scaffold_builder* as a solution to scaffolding pre-assembled contigs against a reference sequence. *Scaffold_builder* enables contigs derived from draft genome sequencing to be sorted and similar contig termini to be merged where the *de novo* assembly program broke the contigs, for example, in a repeat region. While generating draft genomes remains considerably faster and cheaper than generating complete genome sequences, *Scaffold_builder* both increases the value of these drafts by predicting global genomic context, and brings down the cost of gap closure by suggesting targets for PCR validation.

### Availability and requirements

Project name: *Scaffold_builder*

Project and webserver home page: http://edwards.sdsu.edu/scaffold_builder.

Operating system: the program was developed for Linux but should also run on Windows or OS X command line interpreters.

Programming language: Python.

Other requirements: Nucmer (http://mummer.sourceforge.net) and Python programming language (http://www.python.org).

License: GNU GPL3.

Any restrictions to use by non-academics: no special restrictions.

## Competing interests

The authors declare that they have no competing interests.

## Authors’ contributions

GGZS wrote the *Scaffold_builder* script. GGZS, BED, TDM and RAE designed the algorithm. TDM, KE and EAD validated the tool. GGZS, BED, RS, TDM and RAE drafted the manuscript. RAE conceived of the study. All authors read and approved the final manuscript.

## Supplementary Material

Additional file 1: Table S1 Details of scaffolding statistics for real and simulated genomes. Click here for file

Additional file 2: Table S2 Details of the gaps between scaffolded contigs. These details include the locations and lengths of the gaps (negative gap lengths indicate insertions relative to the reference) and identifiers and annotation of any overlapping genes. The file contains 4 tabs: one each for the chromosomes and plasmids of both *S.* Typhimurium strains. Click here for file

Additional file 3: Table S3 Comparison between *Scaffold_builder* and CONTIGuator. Click here for file
